# Childhood psychological abuse influences adolescent aggression through mentalizing and alexithymia sequential mediation

**DOI:** 10.1038/s41598-025-07449-w

**Published:** 2025-07-02

**Authors:** Shuilian Duan, Xizheng Xu, Huaying Pi, Ji Lai, Yuesheng Huang

**Affiliations:** 1 Management Department, Hunan Police Academy, Changsha, 410138 China; 2 College of Education, Hunan First Normal University, Changsha, 410138 China

**Keywords:** Childhood psychological abuse, Mentalizing, Alexithymia, Aggression, Sequential mediating, Psychology, Human behaviour

## Abstract

This study explores the impact of childhood psychological abuse on adolescent aggression and delves into the sequential mediating roles played by mentalizing and alexithymia within this dynamic. Engaging a cohort of 911 middle and high school students in China, the research uncovers that childhood psychological abuse is a significant predictor of aggression in adolescents. It further identifies alexithymia as a mediator in the correlation between childhood psychological abuse and adolescent aggression. Additionally, a sequential mediation by mentalizing and alexithymia elucidates the complex link connecting childhood psychological abuse to aggression. These insights shed light on the psychological underpinnings of aggressive behaviors in adolescents and lay a foundation for crafting targeted prevention and intervention strategies.

## Introduction

Adolescent aggression, defined as intentional behavior aimed at causing physical or psychological harm^1^, poses a significant developmental challenge with long-term repercussions. This maladaptive behavior not only inflicts immediate harm on victims but also predicts adverse outcomes for perpetrators,including an increased risk of depressive disorders (OR = 1.52, 95% CI = 1.27–1.80, $$p < 0.0001$$)^2^. Recent studies estimate the annual economic burden of youth aggression and violence in the U.S. at $122–150 billion, with healthcare and criminal justice costs comprising over 60% of total expenditures^3^. Given these far-reaching consequences, a deeper understanding of aggression’s developmental pathways is crucial.

Among the various risk factors for aggression, childhood maltreatment stands out as particularly consequential. Meta-analytic evidence indicates that approximately 38% of aggressive adolescents report histories of abuse^4^, with psychological abuse (e.g., verbal humiliation, emotional neglect) demonstrating distinct predictive power. While physical abuse is associated with hyperactivity in the amygdala, a key node of the threat-response system^5^, psychological abuse disproportionately disrupts social-cognitive networks. This is evidenced by reduced cortical thickness in the anterior insula and medial prefrontal cortex (PFC)^6^ , deficits in mental state attribution compared to general cognitive control^7^, and increased relational rather than physical aggression^8^.

The General Aggression Model^9^(GAM) provides a comprehensive framework for understanding the development of aggression, highlighting three key mechanisms: affective (heightened negative emotionality), cognitive (hostile attribution biases), and arousal-based (chronic physiological hyperreactivity) pathways. In this study, we examine how mentalization and alexithymia map onto the cognitive and affective pathways of the GAM. Within this framework, alexithymia, which involves difficulties in identifying and describing emotions alongside externally oriented thinking^10^, emerges as a critical mediator linking psychological abuse to aggression. Research suggests that childhood psychological abuse fosters alexithymia by promoting emotional suppression, where abused children learn to avoid processing distressing emotions as a coping mechanism^11^. This avoidance of emotional awareness hinders effective emotional regulation and the development of healthy coping strategies, leading to an increased reliance on maladaptive responses such as aggression. Furthermore, alexithymia impedes the ability to recognize and articulate emotions, exacerbating cognitive distortions like hostile attribution biases, where ambiguous social cues are interpreted as threatening^12^ . Thus, alexithymia plays a dual role in the aggression process: it not only impairs emotional regulation but also distorts social cognition, making individuals more likely to react aggressively in emotionally charged situations^13^. These findings underscore the significance of alexithymia in the pathway from psychological abuse to aggression, offering important insights for interventions aimed at reducing aggressive behavior in individuals with a history of emotional trauma.

Beyond alexithymia, mentalization-the ability to understand one’s own and others’ mental states-has been increasingly implicated in the development of aggression^14^. Notably, emerging research suggests that mentalization propensity (the motivation to consider mental states) may play a more decisive role in aggression than mentalization ability (the cognitive capacity to infer mental states)^15^ . This distinction is crucial, as aggressive individuals often retain the cognitive resources for mentalization but exhibit reduced motivation to apply them in social interactions^7^. Developmentally, exposure to psychological abuse fosters a defensive detachment from mental states, as attending to one’s own or others’ emotions may be distressing in an invalidating environment^16^. This defensive stance manifests as a diminished mentalization propensity, wherein individuals avoid reflective engagement with emotions, leading to impulsive and poorly regulated aggression^17^. Additionally, some individuals may develop hypermentalizing tendencies-excessively attributing complex, often hostile intentions to others-exacerbating relational conflicts and reactive aggression^17^. These findings suggest that childhood psychological abuse may contribute to aggression through dual mentalization-related pathways: a hypo-reflective pathway (low mentalization propensity, resulting in impulsive aggression) and a hyper-reflective pathway (over-interpretation of social cues, fueling hostile attribution biases).The above two mentalization-related pathways are closely linked to mentalization propensity. They essentially reflect different manifestations of mentalization propensity.

Building on these foundations, we propose a developmentally sensitive sequential mediation model that traces the pathway from childhood psychological abuse to adolescent aggression through impairments in mentalizing propensity and subsequent alexithymia. This hypothesized pathway unfolds through three interconnected mechanisms: First, psychological abuse disrupts the early emergence of mentalizing propensity, as evidenced by reduced activation in mentalizing-related neural networks^6^. Second, these deficits in mentalizing propensity increase vulnerability to alexithymia. This sequential ordering is theoretically justified because mentalizing-the capacity to understand mental states in self and others-is developmentally antecedent to and necessary for emotional awareness. The ability to recognize and represent one’s own mental states is prerequisite for identifying and articulating emotions; when this foundation is compromised, alexithymia naturally follows. Longitudinal evidence supports this developmental sequence, with mentalizing impairments at age 5 predicting alexithymia at age 8^13^.

The relationship between these constructs is nuanced; alexithymia’s association with mentalizing varies across dimensions (ability vs. propensity, emotional vs. non-emotional). Specifically, alexithymia correlates strongly with deficits in emotionally-laden mentalizing tasks but shows weaker associations with non-emotional mental state understanding. Self-report measures consistently reveal reduced mentalizing propensity among individuals with alexithymia^15^. Given our cross-sectional design, this theoretically-driven sequential ordering provides the conceptual framework for our mediation analysis. Finally, alexithymia facilitates aggression by fostering undifferentiated physiological tension, impairing emotion regulation, and encouraging externalizing responses to distress when internal emotional states cannot be effectively processed^11,12^.

**Fig. 1:**
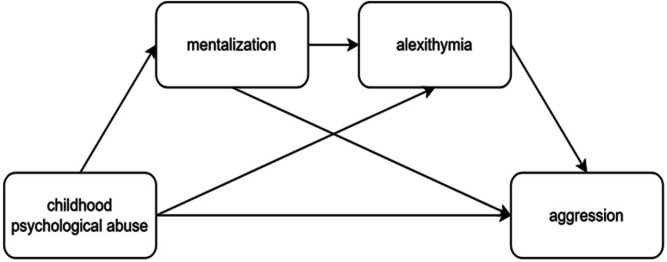
The hypotheses model of study.

Despite increasing interest in these relationships, existing research suffers from three critical limitations: (1) lack of developmental temporal sequencing, which overlooks key developmental windows; and (2) isolated mechanisms, with affective and cognitive pathways examined separately rather than in an integrated framework. To address these gaps, the present study proposes a Cognition-Affect Sequential Developmental Model. Using a cross-sectional design, we test the following hypotheses:

- H1: Childhood psychological maltreatment significantly predicts adolescent aggression.
- H2: Mentalizing propensity mediates the relationship between psychological maltreatment and aggression.
- H3: Alexithymia also functions as an independent mediator in this relationship.
- H4: A significant sequential mediation pathway exists: childhood psychological maltreatment $\rightarrow$ mentalizing propensity $\rightarrow$ alexithymia $\rightarrow$ aggression. Figure 1 shown the frame model of the above four hypotheses.

By integrating cognitive and affective mechanisms into a sequential mediation framework, this study aims to refine theoretical models of aggression and offer insights into the developmental impact of childhood psychological maltreatment, particularly emotional abuse-findings that have important implications for targeted intervention approaches.

## Method

### Ethics approval

This study received approval from the Ethics Review Committee of Hunan First Normal University. All procedures involving human participants adhered to the ethical guidelines set forth in the Declaration of Helsinki. Participants were informed about the study’s purpose, guaranteed the confidentiality of their responses, and notified of their right to withdraw at any time. Informed consent was obtained electronically from all participants before data collection commenced.

### Participants

This study employed a stratified random sampling method to recruit 911 students from five middle and high schools across Hunan, Yunnan, and Hainan provinces. After excluding 184 invalid or incomplete questionnaires, 727 valid responses were analyzed, resulting in an effective response rate of 79.8%. The final sample comprised 350 male students (48.14%) and 377 female students (51.86%), with a diverse distribution across grades: 63 first-year, 56 second-year, and 223 third-year middle school students; and 158 first-year, 128 second-year, and 99 third-year high school students, with an average age of 15. All participants were Mandarin-speaking Han Chinese. This cross-sectional study used retrospective self-report measures, with data collected from September to December 2022. Participants were asked to recall childhood experiences, with an average of 5.2 years since exposure to abuse (SD = 2.1). While memory accuracy may be influenced by various factors^18,19^, meta-analytic evidence suggests that childhood trauma recall is moderately stable over the long term (ICC = 0.56–0.72)^20^.

### Instruments

#### Measurement of childhood psychological abuse

The “Psychological Abuse Subscale” of the the Childhood Trauma Questionnaire (CTQ), developed by Bifulco et al^21^, was employed to measure experiences of psychological abuse. This subscale, validated for its reliability and consisting of scolding intimidation, and interference factors, utilizes a 5-point Likert scale from “0 = never” to “4 = always”, where a higher cumulative score signifies more severe experiences of childhood psychological abuse. The Cronbach’s alpha in this study was 0.90.

#### Measurement of mentalizing propensity

Following Fonagy et al.^14^, we conceptualized mentalizing as the self-reported propensity to reflect on one’s own and others’ mental states (rather than objective ability).We measured individuals’ self-reported tendency to engage with mental states using the 46-item Reflective Function Questionnaire for Youth (RFQ-Y). This scale specifically evaluates propensity rather than objective ability. Participants rated each item on a 6-point Likert scale, ranging from “strongly agree” to “strongly disagree,” with higher scores indicating greater reflective functioning. In this study, the Cronbach’s alpha for the scale was 0.88, indicating good internal consistency.

#### Measurement of alexithymia

The Toronto Alexithymia Scale (TAS-20), developed by Bagby et al^22^, was utilized to assess alexithymia across four dimensions: identifying emotions, describing emotions, differentiating emotions from bodily sensations, and external-oriented thinking. This 20-item scale employs a 5-point Likert scale, with higher scores reflecting greater levels of alexithymia. In this study, the Cronbach’s alpha for the scale was 0.83.

#### Measurement of aggression

Aggression was assessed using the Buss-Perry Aggression Questionnaire(BPAQ)^23^, which consists of 29 items that evaluate four dimensions of aggression: physical aggression, verbal aggression, anger, and hostility. Each item is rated on a 5-point Likert scale, ranging from 1 (“strongly disagree”) to 5 (“strongly agree”). with higher scores indicating higher levels of aggression. This scale has demonstrated strong reliability, with a Cronbach’s alpha of 0.89.

### Procedure

Faculty shared an ethics-approved invitation via WeChat class groups. Interested students accessed the survey link. The survey, conducted on Wenjuanxing, included demographic questions and psychological measures. Participants could proceed only after consenting. Responses were anonymous, with no personal identifiers or IP addresses collected.

### Data analysis

Prior to analysis, data were screened for violations of statistical assumptions. Continuous variables were examined for normality using skewness (<|2|) and kurtosis (<|7|) thresholds^24^. Preliminary analyses included descriptive statistics (means, standard deviations) and bivariate Pearson correlations among all study variables. Multicollinearity was assessed through variance inflation factors (VIF), with all values below 2.5 indicating no substantial concerns. Missing data (3.2% of cases) were handled using full information maximum likelihood estimation (FIML), Little’s MCAR test indicated no evidence against completely random missingness ($\lambda ^2$=5.32, df=8, $p=0.26$), suggesting that the stricter Missing At Random (MAR) assumption required by FIML is plausible. Although FIML provides more efficient estimates than listwise deletion under MAR, we note that potential violations (e.g., Missing Not At Random) could still bias results^25^.

To examine the hypothesized chain mediation model, we conducted mediation analyses using Hayes’ (2018) PROCESS macro (Model 6) in SPSS^26^. This approach employs ordinary least squares (OLS) regression-based path analysis combined with bias-corrected bootstrapping (5,000 resamples) to estimate indirect effects. Unlike traditional Baron & Kenny causal steps approach^27^, PROCESS directly tests the product of coefficients (a $\times$ b) for mediation effects. If the 95% bootstrap confidence interval (CI) for an indirect effect does not include zero, the mediation is considered significant. Note that while PROCESS improves upon classical methods by accounting for non-normal sampling distributions, it does not adjust for unmeasured confounding^28^ (cf. causal mediation analysis frameworks). Thus, findings represent statistical associations rather than causal effects unless supported by experimental/longitudinal designs. All statistical models adjusted for two key demographic confounders: Age (continuous, in years) Gender (binary: 0 = male, 1 = female). Detailed output interpretation guidelines are provided in Results.

## Results

### Common method bias test

The evaluation of common method bias was conducted using Harman’s single-factor test^29^. The analysis identified ten factors with eigenvalues exceeding 1, with the variance attributed to the primary factor being only 10.33%. This figure is significantly lower than the commonly accepted threshold of 40%, indicating that common method bias is unlikely to compromise the dataset’s integrity.

### Descriptive analyses

Table 1’s correlation analysis reveals key relationships between childhood psychological abuse, aggression, mentalization, and alexithymia. Childhood psychological abuse showed significantly correlation with aggression ($r = 0.36$, a medium effect according to Cohen, 1988)^30^. Childhood psychological abuse negatively correlated with mentalization, indicating that abuse might impair the understanding of mental states. Aggression correlates positively with psychological abuse and alexithymia, showing that more abuse and emotional expression difficulties can lead to increased aggression. There’s no significant link between aggression and mentalization, hinting at a complex relationship. Alexithymia is positively related to psychological abuse and aggression but negatively to mentalization, emphasizing its role in linking abuse experiences with aggression and reduced mentalization.

**Table 1:** Bivariate correlations among study variables.

	M	SD	1	2	3	4	5
1 Age	15	1.36	–				
2 Sex	–	–	0.01	–			
3 Abuse	0.87	0.59	0.01	0.03	–		
4 Aggression	75.39	14.35	0.02	0.08	0.361**	–	
5 Alexithymia	72.05	10.17	0.02	0.07	0.253**	0.315**	–
6 Mentalization	8.52	0.87	0.07	0.06	$-0.111$**	$-0.072$	$-0.186$**

**$$p < 0.01$$.

### The mediation model of psychological abuse and aggression

The foundational insights from the preliminary correlation analysis set the stage for a deeper investigation into the mediating roles of mentalization and alexithymia. The mediation analysis uncovered significant pathways linking childhood psychological abuse to mentalization, alexithymia, and subsequently aggression. Childhood psychological abuse emerged as a significant predictor of increased aggression in adolescents, indicating a direct correlation between higher instances of reported abuse and elevated aggression. Furthermore, psychological abuse was found to negatively affect mentalization, implying that abuse undermines the ability to interpret and understand mental states—both one’s own and those of others. In contrast, psychological abuse positively correlated with alexithymia, suggesting that abusive experiences intensify difficulties in identifying and expressing emotions. A noteworthy negative relationship was observed between mentalization and alexithymia, revealing that deficits in mentalization are associated with heightened alexithymic symptoms. Alexithymia significantly predicted increased aggression, even when accounting for the effect of psychological abuse, which remained a significant aggression predictor.

**Fig. 2:**
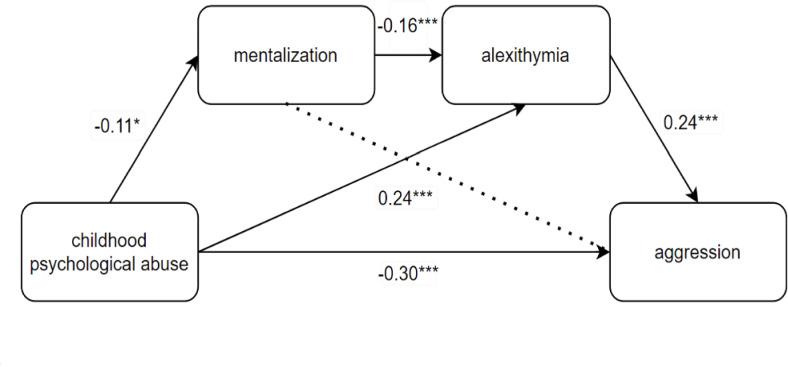
Chain-mediated graph of the effect of childhood psychological abuse on aggression. *$p < 0.05$; ***$p < 0.001$. Dashed lines represent insignificant paths.

While the alexithymia-only and sequential mentalization-to-alexithymia pathways showed significant indirect effects, the mentalization-only path was not significant (effect size = $-0.026$, 95% CI [$-0.043, 0.006$]). Table 2 presents the parameter estimates of the mediation model. The validation of our hypothesis model is presented in Fig. 2.

**Table 2:** Direct, indirect, and total effects from the chain mediation model.

Path	ES	SE	BootLLCI	BootULCI
Psychological abuse$\rightarrow$mentalization$\rightarrow$aggression	$-0.026$	0.005	$-0.043$	0.006
Psychological abuse$\rightarrow$alexithymia$\rightarrow$aggression	0.058	0.012	0.035	0.081
Psychological abuse$\rightarrow$mentalization$\rightarrow$alexithymia$\rightarrow$aggression	0.004	0.002	0.001	0.009
Psychological abuse$\rightarrow$ aggression	0.024	0.005	$-0.041$	0.006
Total indirect effects	0.060	0.012	0.038	0.084

The ES represent indirect effects obtained from the regression analysis. Boot LLCI and Boot ULCI denote the lower and upper limits of the 95% confidence intervals obtained from the 5000-sample Bootstrap procedure

Results supported Hypothesis 3 and Hypothesis 4. However, Hypothesis 2 was not statistically supported.

## Discussion

This study sheds light on the intricate relationship between childhood psychological abuse and adolescent aggression, highlighting the significant mediating roles of alexithymia and mentalization propensity. Our findings provide empirical support for a complex developmental pathway from early emotional trauma to later behavioral difficulties, offering valuable insights for both theoretical frameworks and clinical interventions.

The direct association between childhood psychological maltreatment and heightened aggression in adolescence validates our first hypothesis and aligns with established theoretical perspectives. The observed correlation between childhood abuse and aggression ($r = 0.36$) aligns with meta-analytic estimates ($r = 0.32-0.38$).^4^ This finding aligns with contemporary evidence that aggression may function as a compensatory response to early socioemotional deprivation^31^, supported by shared neural circuitry between social rejection and aggressive motivation^32^. Furthermore, consistent with Bandura’s social learning theory of aggression^33^, childhood psychological abuse may establish maladaptive behavioral schemas through observational learning processes. Attachment theory further elucidates how early emotional abuse fosters maladaptive cognitive schemas^34^, such as self-deprecation, interpersonal mistrust, and hostile attributional biases in ambiguous social interactions. This theoretical convergence underscores the urgency of preventing both overt and subtle forms of emotional maltreatment while cultivating emotionally attuned, respectful, and supportive environments to mitigate aggressive tendencies in adolescence.

Our analysis confirms alexithymia as a significant mediator in the relationship between childhood psychological abuse and adolescent aggression, supporting our second hypothesis. This suggests that deficits in emotional processing—specifically, difficulties in identifying and articulating emotions-serve as a crucial mechanism linking early emotional trauma to later aggression. While the specific combination of childhood emotional abuse, alexithymia, and aggression has received limited empirical attention, our results align with prior research on their pairwise relationships^35^. The observed pattern supports the notion of hyperalexithymia as a developmental consequence of emotionally invalidating parental responses and insecure attachment formations. These findings underscore the potential efficacy of interventions aimed at enhancing emotional awareness and expression skills as a strategic approach to reducing aggression among adolescents with histories of emotional abuse.

The results concerning mentalization propensity (distinct from mentalization ability) require nuanced interpretation. Our findings extend previous research by Pisani et al. on emotion recognition deficits in alexithymia^15^. Rather than merely documenting impairments in emotion-specific processing, our data suggest a broader disruption-a diminished willingness to engage with mental states altogether. This points to a metacognitive deficit in mentalization propensity that extends beyond emotions to affect reflective functioning more generally. However, our exclusive reliance on self-report measures of mentalization propensity (RFQ-Y) represents a methodological limitation, restricting direct comparability with performance-based mentalization ability studies. Future research employing multimodal assessments could offer a more comprehensive perspective on the cognitive-affective disruptions associated with childhood emotional abuse.

Contrary to theoretical expectations^36^ and previous clinical observations^37^, mentalization propensity alone did not significantly mediate the emotional abuse-aggression pathway. This unexpected result may reflect several important nuances. First, emotional abuse may selectively impair specific dimensions of mentalization propensity rather than the general propensity measured by the RFQ-Y. This aligns with neuroimaging findings that demonstrate distinct neural substrates for emotional versus cognitive mentalization processes^38^. Second, the mentalization propensity-aggression relationship may be moderated by relational context, with secure attachment relationships potentially buffering aggressive tendencies despite mentalization difficulties^3^. Our study did not assess attachment histories, leaving this possibility unexplored. Third, the RFQ-Y’s focus on conscious self-perception of mentalization may inadequately capture implicit mentalization tendencies, which might more reliably predict behavioral outcomes^39^. This highlights the “knowing-doing gap” in mentalization research, warranting further investigation.

Despite the limited individual mediating effect of mentalization propensity, the significant combined mediation through mentalization propensity and alexithymia delineates a crucial sequential pathway connecting childhood emotional abuse to adolescent aggression. This chain mediation model clarifies how early emotional trauma disrupts cognitive-affective development through complementary mechanisms: reduced mentalization propensity contributes to alexithymic difficulties, which in turn foster aggressive behavioral responses^40^ . These findings suggest that interventions targeting both mentalization enhancement and emotional processing skills may offer the most effective approach for mitigating aggression among adolescents with histories of psychological abuse^41^. Mentalization-based treatments that explicitly focus on fostering the propensity to mentalize within emotional contexts may be particularly beneficial for this population.

The sequential mediation pathway (psychological abuse $\rightarrow$ impaired mentalizing $\rightarrow$ elevated alexithymia $\rightarrow$ increased aggression) accounted for 19% of the total variance, providing empirical validation for our Cognition-Affect Cascade Model. This finding extends the General Aggression Model (GAM) by demonstrating that mentalizing impairments (representing the cognitive pathway) temporally precede and contribute to the development of alexithymia (affective pathway), replicating developmental sequences documented in normative populations. Notably, the non-significant direct mediation through mentalizing alone stands in contrast to the hypermentalizing patterns observed in borderline personality disorder, indicating that childhood psychological abuse may predominantly diminish the motivation for reflective processing (mentalizing propensity) without necessarily impairing the accuracy of mental state attribution.

### Implications for practice and policy

The findings highlight two key practical implications. First, interventions for aggression in abused adolescents should adopt a staged approach, initially enhancing mentalizing capacity through reflective exercises before addressing emotional awareness to mitigate alexithymia. This sequenced strategy may prove more effective than isolated cognitive or affective interventions. Second, the results advocate for trauma-informed screening in schools to identify at-risk youth. Brief assessments of mentalizing and emotional awareness could facilitate early intervention through school-based programs combining mentalization strategies and emotion regulation training, increasing accessibility while reducing stigma. These findings support allocating resources to integrate such targeted components into existing anti-aggression initiatives and providing educator training to recognize early warning signs.

### Limitation and future direction

Taken together, the study contributes to applied knowledge across clinical, educational, and policy domains; however, its limitations must be considered to contextualize these implications. First, the cross-sectional design limits causal inference. Although our model is theoretically grounded, alternative explanations, such as genetic factors (e.g., 5-hydroxytryptamine transporter gene polymorphisms), may affect both maltreatment exposure and emotional processing. Therefore, our findings should be viewed as hypotheses for future longitudinal and experimental research.^42^ Second, methodological challenges arise in three areas: (1) Measurement- the reliance on retrospective self-reports may introduce recall bias, as participants’ recollections of childhood experiences could be influenced by current psychological states; future studies should include multi-informant data (e.g., parent/teacher reports) and biological indicators like neuroendocrine measures. (2) Sample-the study’s cultural context may limit generalizability, underscoring the need for cross-cultural research on maltreatment’s developmental impact. (3) Confounding-beyond age and gender, other factors such as socioeconomic status and epigenetics should be accounted for. Future work should use longitudinal designs to track how alexithymia and mentalization contribute to aggression over time, while testing potential moderators (e.g., attachment, peer relationships) and mediators such as hostile attribution bias.^43,44^ Neurobiological tools (e.g., fMRI, cortisol) could clarify underlying mechanisms. A multi-level approach will improve our understanding of the long-term effects of emotional abuse and inform effective interventions.

## Conclusion

This study illuminates the complex mechanisms linking childhood psychological abuse to adolescent aggression. The findings confirm that childhood psychological abuse significantly predicts increased aggressive behavior in adolescents. While mentalization alone did not demonstrate a significant mediating effect, alexithymia emerged as a crucial mediator in this relationship. Furthermore, the research revealed a significant chain mediation effect, where psychological abuse influences aggression through the sequential pathway of impaired mentalization and increased alexithymia.

## Data Availability

The datasets generated and/or analyzed during the current study are available from the corresponding author on reasonable request.
